# The InterMine Android app: Cross-organism genomic data in your pocket

**DOI:** 10.12688/f1000research.17005.2

**Published:** 2019-06-07

**Authors:** Daria Komkova, Rachel Lyne, Julie Sullivan, Yo Yehudi, Gos Micklem

**Affiliations:** 1 Department of Genetics, University of Cambridge, Downing Street, Cambridge, CB2 3EH, UK

**Keywords:** Android app, Genomics data, Gene search, InterMine

## Abstract

InterMine is a data integration and analysis software system that has been used to create both inter-connected and stand-alone biological databases for the analysis of large and complex biological data sets. Together, the InterMine databases provide access to extensive data across multiple organisms. To provide more convenient access to these data from Android mobile devices, we have developed the InterMine app, an application that can be run on any Android mobile phone or tablet. The InterMine app provides a single interface for data access, search and exploration of the InterMine databases. It can be used to retrieve information on genes and gene lists, and their relatives across species. Simple searches can be used to access a range of data about a specific gene, while links to the InterMine databases provide access to more detailed report pages and gene list analysis tools. The InterMine app thus facilitates rapid exploration of genes across multiple organisms and kinds of data.

## Introduction

InterMine ^1^ is an open source data integration and analysis software system (license LGPL 2.1) that has been used to create a suite of both inter-connected and stand-alone biological databases for the analysis of large and complex biological data sets. InterMine databases have been developed for the major model organisms budding yeast ^2^, nematode worm, fruit fly ^3^, zebrafish, mouse ^4^ and rat ^5^, (which we will refer to as the Model Organism Database (MOD-) InterMines, together with a human database and databases for plants, bees and wasps ^6^, cows ^7^, *Medicago truncatula* ^8^, mitochondrial proteomics ^9^ and drug targets ^10^ ( Table 1; https://registry.intermine.org/). Together, the InterMine databases provide access to extensive data across multiple organisms (for full listings of data included see the website for each individual InterMine, Table 1). To provide more convenient access to these data from Android mobile devices, we have developed the InterMine app ^11^, an application that can be run on any Android mobile phone or tablet.

**Table 1.** InterMine databases available through the InterMine app by default. The list can be configured in the app.

Database	Data	Organisation	url
FlyMine	Fruit fly	InterMine	http://www.flymine.org
HumanMine	Human	InterMine	http://www.humanmine.org
MedicMine	*Medicago* *truncatula*	JCVI	http://medicmine.jcvi.org
MouseMine	MGI	MGI	http://www.mousemine.org
PhytoMine	Multiple plant genomes	Phytozome	https://phytozome.jgi.doe.gov
RatMine	Rat	RGD	http://ratmine.mcw.edu
TargetMine	Support for drug discovery	NIBIO	http://targetmine.mizuguchilab.org
ThaleMine	Thale cress	Araport	https://apps.araport.org/thalemine
WormMine	Nematode	WormBase	http://intermine.wormbase.org/tools/wormmine
YeastMine	Budding yeast	SGD	http://yeastmine.yeastgenome.org/yeastmine
ZebrafishMine	Zebrafish	ZFIN	http://zebrafishmine.org/
HymenopteraMine	Various Hymenoptera	University of Missouri	http://hymenopteragenome.org/hymenopteramine
BovineMine	Bos taurus	Bovine Genome Database Project	http://bovinegenome.org/bovinemine

The InterMine app provides a single interface for data access, search and exploration of the above databases. It can be used to retrieve information on genes and gene lists, and their relatives across species. Simple searches can be used to access a range of data about a specific gene, while links to the InterMine databases provide access to more detailed report pages and gene list analysis tools. Although a number of mobile applications have been developed for the laboratory (see https://www.biocompare.com/Editorial-Articles/168745-Ten-Mobile-Apps-for-Biology-Laboratories/), only a few so far exist for biological databases. Some examples include the YeastGenome app developed by SGD ^12^, Molecules, for viewing 3D protein structures ( https://itunes.apple.com/us/app/molecules/id284943090?mt=8) and Pubmed on Tap ( https://itunes.apple.com/us/app/pubmed-on-tap/id301316540?mt=8) / Pubmed Mobile ( https://play.google.com/store/apps/details?id=com.bim.pubmed&hl=en) for searching Pubmed and retrieving PDFs. Thus, development of the InterMine app was largely an exploratory exercise as it was not known at the outset what sort of demand there would be for accessing data in such a way, although we were encouraged by the success of the yeastGenome app. It is intended that the app provides quick and easy access to information about Genes when researchers may be away from their main computing source, such as when attending a conference or meeting. However, in addition to providing a quick and novel way to access biological data, InterMine app also expands InterMine’s functionality in allowing all registered InterMine databases to be searched at once, thus providing a cross-organism view of the term(s) searched.

The app is available from the Google Play Store at https://play.google.com/store/apps/details?id=org.intermine.app .

## Methods

### Data sources

The InterMine app allows users to search a default subset of InterMine data warehouses ( Table 1). This list is configurable, and so users are able to refine or add mines to match their interests. See https://registry.intermine.org for the full list of known public InterMine instances.

InterMine databases typically integrate data from many resources. For instance BioGRID ^13^, IntAct ^14^, UniProt ^15^, and can include high quality curated data (from the Model Organism Databases), genome-wide high-throughput data and data from smaller more focused studies (See individual InterMine websites for more details). All the InterMine databases accessible from the app are open source (License LGPL 2.1) and the data within them is free to access and download. Some individual InterMines may have restrictions for commercial use of the data and each individual InterMine should be consulted for its policy. See Table 1 for URLs.

### Search and analysis

The InterMine app provides several ways to search and explore the data available, including a keyword search, sets of pre-defined template searches and list analysis functionality. These features are described in more detail in the use case section.

### MyMine accounts

InterMine databases allow users to create an account through which they can, between sessions, store lists and searches. The InterMine App therefore allows users to log in to any accounts they hold on the underlying databases, so enabling user-created lists to be accessed.

### Favourite genes

Users are also able to mark genes in search results as *favourite*. These genes are stored on the Android device and can be accessed without needing to log in to any of the underlying databases.

## Implementation

### Communication

The InterMine database design and the webservices used to power the InterMine app have been previously described ^1, 16^. The InterMine App draws all data from the RESTful Application Programming Interfaces (APIs) that InterMine databases provide ^16^. RoboSpice, an Open Source (Apache 2.0 Licence) Android communications library ^17^, provides core network communication functionality. Data are loaded asynchronously over HTTP or HTTPS, depending on the preferred protocol of the database being accessed. For performance enhancement, most web service responses are stored on the device if headers state an appropriate cache lifetime.

When the app receives a JSON response from the web service, it transforms the data from a table-structured format to a more hierarchical view, which presents data more effectively on smaller-screened mobile devices.

### Authentication

Each InterMine database is discrete and often maintained by different organisations. If a user wishes to authenticate with multiple InterMines - perhaps to view private gene lists stored on different databases - they will need to provide separate authentication details for each InterMine database. However this is only necessary once, as after a user has successfully authenticated in a given InterMine via a username/password pair, the app retrieves and stores an API authentication token. This ensures that the user can authenticate in the future without having to re-enter or store sensitive password details.

All of the user configuration settings and authentication tokens are stored locally on the device via SharedPreferences, Android’s dedicated settings storage mechanism ^18^.

### Internal storage

Tabular data, such as favourite genes within the app, are not suited to the key/value pair storage used in SharedPreferences ^19^, and therefore are stored within an SQLite database on the user’s device. Data stored include the InterMine instance the data originated from, the (e.g.) gene’s identifiers, description, organism, and genomic coordinates.

### Search

Keyword search is available across lists, templates and gene search results. Search results from different databases are presented to the user as a single result set, sorted by the search relevance score generated by each originating database. Search results can be shared via email, instant messaging, and other sharing media in text format, using Android’s ACTION_SEND Intent functionality ^20^. Further data export options are available through links to the relevant full InterMine database instance.

### Advanced information

InterMine also includes advanced analysis tools - particularly data visualisations - which may not be available via the API. To access the extended information about genes or gene lists, a user can load InterMine’s advanced report pages within the app itself. This is implemented via Android’s WebView ^21^ functionality which allows live web pages to be embedded in an application (for example, Figure 2 shows an example of an embedded InterMine WebView).

## Operation

The app is implemented in accordance with Google Material Design ^22^ guidelines, providing a predictable environment for the user, and also supports Android version 4.0 and above, ensuring it is able to run on over 99% of active Android phones as of November 2018 ^23^.

## Use case

The following use case introduces each of the three key features of the InterMine app; Gene search, Templates and Lists with an example of their use. Further details of these InterMine features have been fully described in previous publications from several InterMine databases ^1– 3, 5, 6, 8, 9^.

### Cross-organism gene search

The keyword search simultaneously searches all InterMine databases selected through the *settings* option. Thus, a cross-organism overview of data available for further investigation is provided. Link-outs from the search results to each originating InterMine database provide access to detailed gene report pages. These pages collate information integrated for that gene and typically include functional summaries, ontology annotation, pathway, expression, interaction and disease data and links to additional related data.

**Figure 1.**
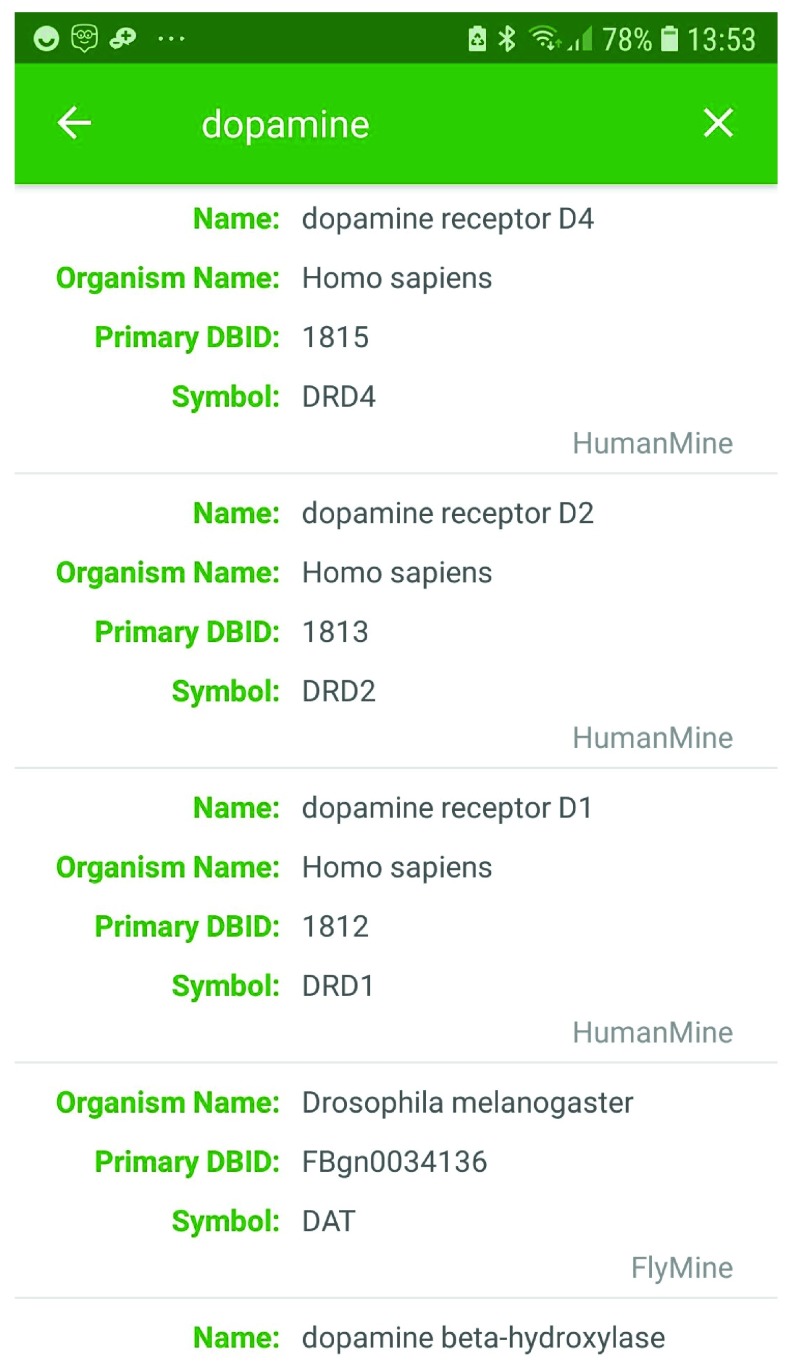
Example keyword search results. A search for dopamine returns genes from PhytoMine, MouseMine, HumanMine, FlyMine, TargetMine, RatMine, ZebrafishMine, YeastMine and ThaleMine. A section of the results from HumanMine and FlyMine is shown.

**Figure 2.**
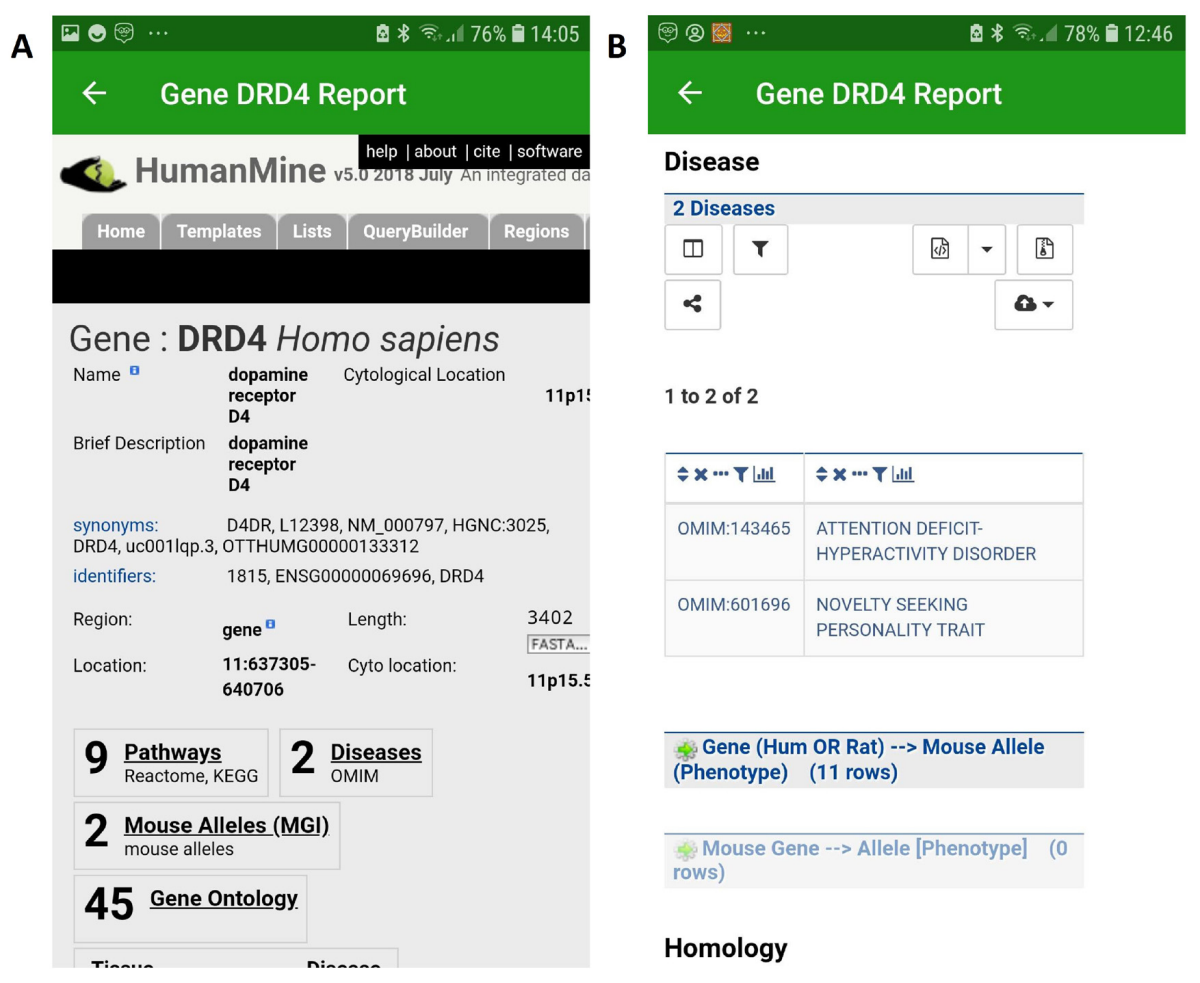
The human DRD4 report page from the HumanMine webapp, displayed in the InterMine app. ( **a**) This view shows the first part of the report page with a summary of the Gene identifiers and data available. ( **b**) This view shows the Disease section of the report page.

As an example, searching for ‘ *dopamine*’ returns dopamine-related genes from PhytoMine, MouseMine, HumanMine, TargetMine, FlyMine, RatMine, ZebrafishMine, WormMine, YeastMine, ThaleMine and HymenoperaMine ( Figure 1; see Table 1 for urls). Selecting a gene from the results, for instance the human gene *DRD4* (dopamine receptor D4) displays summary information about the gene, with a link to the full gene report available from the HumanMine database. Here we learn, for example, that polymorphisms in the *DRD4* gene are associated with the disorder attention deficit hyperactivity disorder (ADHD) ( Figure 2), a condition associated with low dopamine levels. The search results therefore facilitate rapid exploration across multiple organisms and kinds of data.

### Template searches

**Figure 3.**
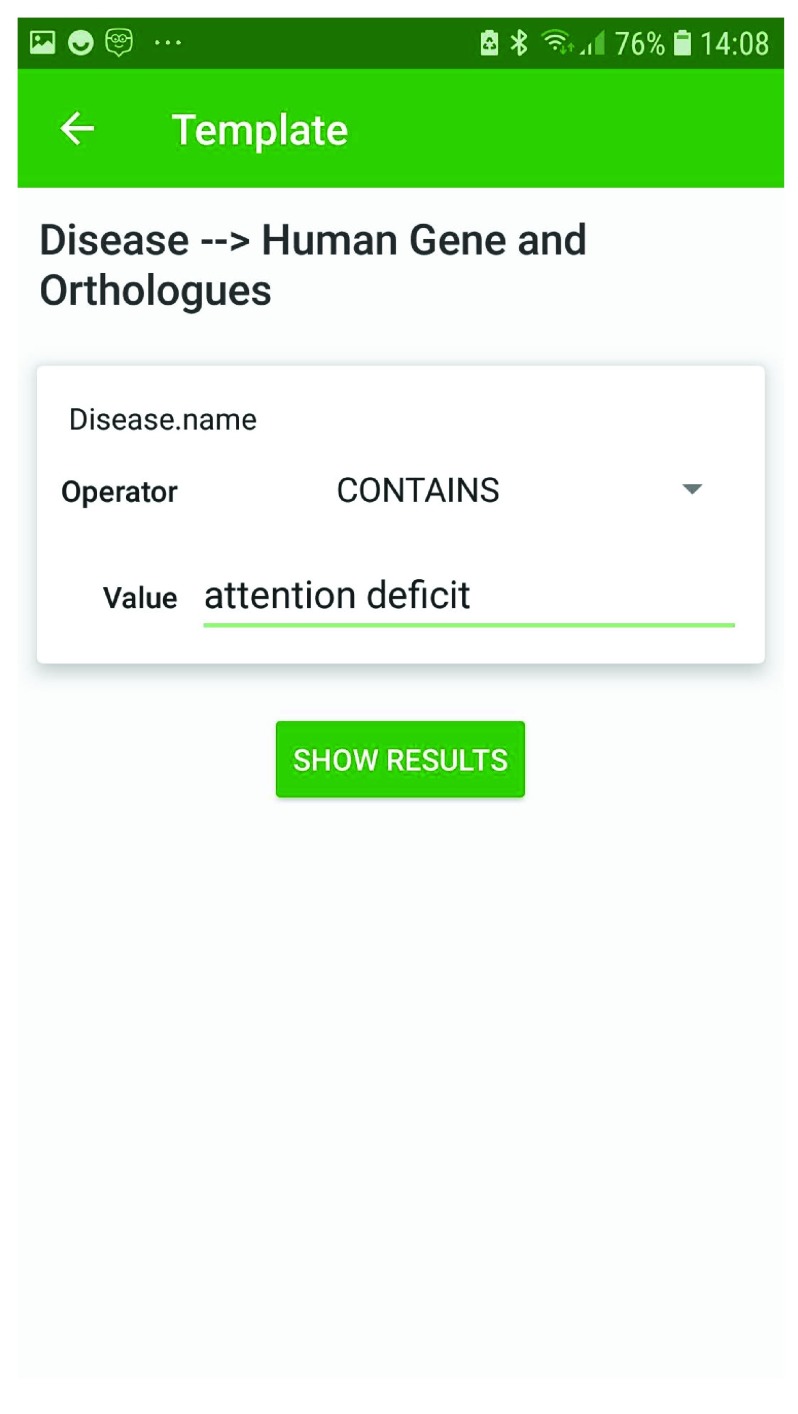
A FlyMine template search, “Disease -> Human genes and Orthologues”. The disease name is an editable constraint, in this case set to search for human genes associated with diseases with a name that “contains” *Attention deficit*, as well as their orthologues.

**Figure 4.**
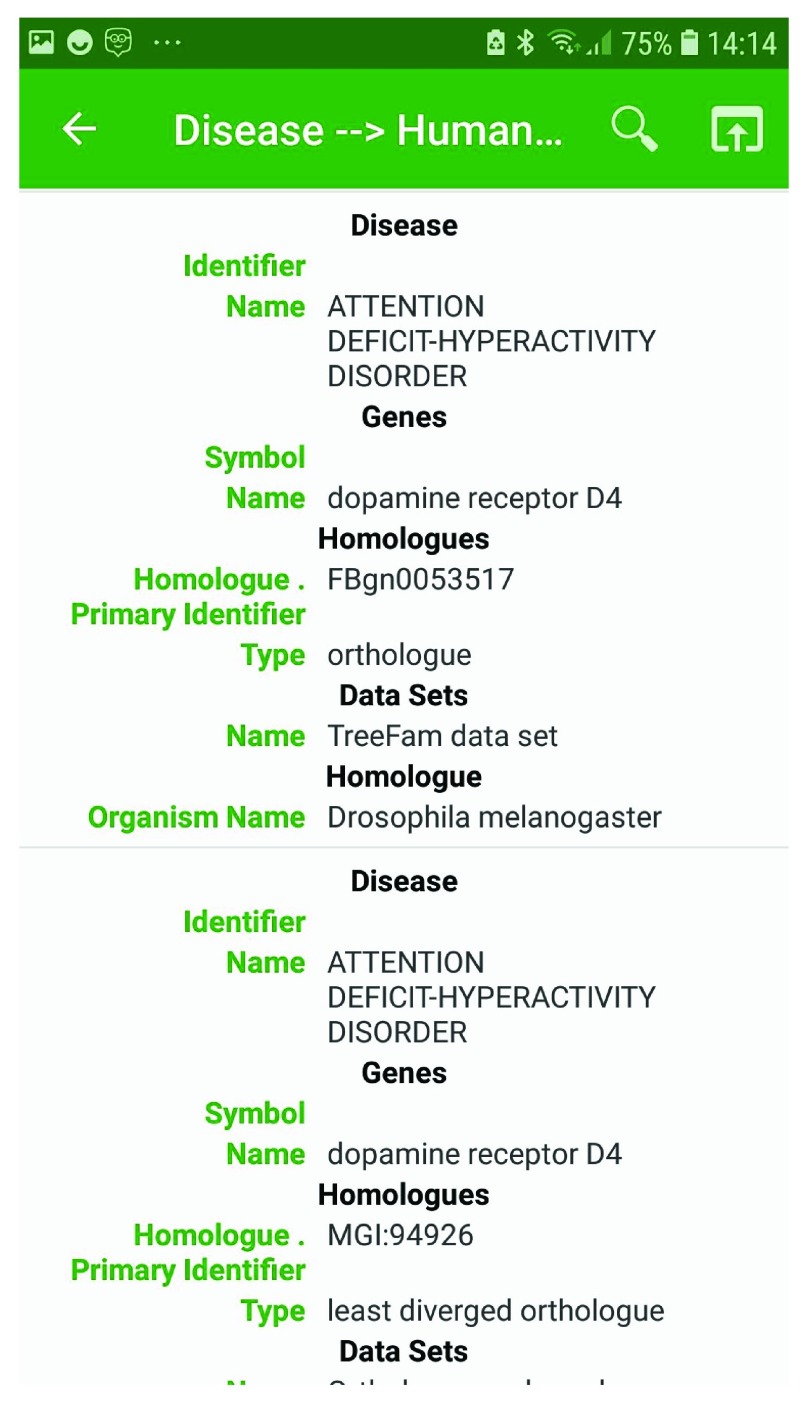
Results from the template “Disease -> Human genes and Orthologues”, with disease constraint set to “contains” Attention deficit, available from FlyMine.

In addition to cross-organism gene search, the InterMine databases provide libraries of pre-built searches, called *template searches*. Such searches provide a user-friendly interface where the parameters for search filters can be specified. These *templates* range from simple searches, such as *for a specified gene (or genes), return the corresponding Gene Ontology terms* (represented as “ *Gene → Gene Ontology terms”*), to more complex searches combining data of more than one type, such as “ *Tissue + interaction → genes*”, which returns any genes expressed in the specified tissue that also interact with the product of a specified gene. Templates from each InterMine database are available within the InterMine App. The results are provided with a simple keyword search to facilitate further data browsing. For instance, continuing the above dopamine example, we can use a template search to identify genes in *Drosophila* associated with ADHD: on the templates page for the FlyMine database, we find the template “Disease -> Human genes and Orthologues” ( Figure 3). This template allows one to specify disease names that *contains “attention deficit”*, and on running the template, this returns the disease *Attention deficit-hyperactivity disorder* along with associated human genes and their orthologues from the available InterMine databases. Using the ability to search within the results we are able to verify that the human gene we are interested in (DRD4) is associated with this condition, and that this gene has a predicted orthologue in *Drosophila melanogaster, FBgn0053517*, *Dop2R* ( Figure 4). Through such iterative searching we can continue our investigation of this fly orthologue to identify, for instance, interacting partners, pathway and Gene Ontology annotations.

Shows the human *dopamine receptor D4* associated with the disease *attention deficit-hyperactivity disorder* and with the *D. melanogaster* orthologue *FBgn0053517, Dop2R.*

### Lists

InterMine databases are especially suited to the analysis of lists of genes or other entities. Users can create their own lists, which can be stored between sessions if the user has an account for the relevant InterMine database. Again, direct links from lists to the underlying InterMine databases provide access to list analysis tools, for instance enrichment statistics that help identify surprising properties, such as publications that cite an unexpectedly large number of the list members, or GO terms or protein domains that are associated with an unexpectedly large number of list members.

**Figure 5.**
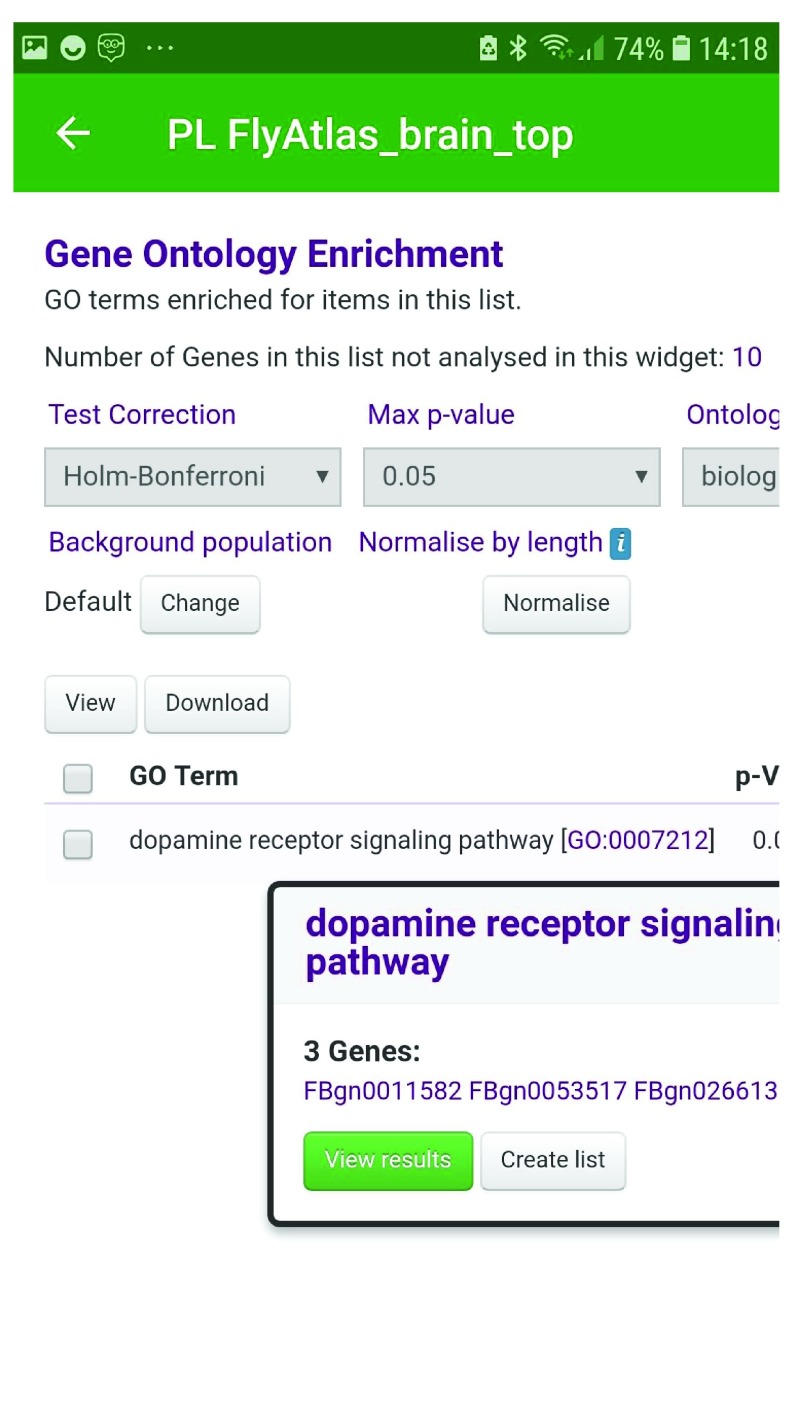
Gene Ontology enrichment analysis of the FlyMine public list PL FlyAtlas_brain_top. A set of three genes (FBgn0011582 (Dpo1R1), FBgn0053517 (dop2R) and FBgn0266137 (Dop1R2) are enriched for the GO term dopamine receptor signaling pathway.

Public lists, which are typically interesting sets of genes derived from publications and other studies, are often provided by the database operators. For instance, in FlyMine, eleven of the public lists provide sets of genes whose expression increases at defined times during drosophila embryogenesis, as derived from Hooper *et al*. ^24^. Further lists show genes that are expressed at increased levels in various adult fly tissues according to data from the FlyAtlas resource ^25^. Within one of these sets, *PL FlyAtlas_brain_top*, we can identify a set of genes up-regulated in the brain. Checking within this list, we find that the dopamine receptor gene *Dop2R* (FBgn0053517) identified above is present. By following the link to the corresponding list analysis page on the underlying FlyMine website, and examining the enrichment statistics, we find that the *Dop2R* gene is part of a set in which the Gene Ontology term *dopamine receptor signaling pathway* (GO:0007212) occurs unexpectedly frequently (p-value 0.001303, with Holm-Bonferroni correction). It is also apparent that two other fly dopamine receptors, *Dop1R1* (FBgn0011582) and *Dop1R2* (FBgn0266137) are also found in this list ( Figure 5).

## Conclusions

The InterMine app provides a convenient way of searching for biological information across many model organism and other databases, allowing an overview of gene function and gene relationships to be pursued. Importantly, the InterMine app reduces the effort required to obtain data available in a range of InterMine databases by removing the need to visit each one individually. Further development of the app is planned, including a single sign-in for all of the InterMine instances through oAuth2 ; further search and analysis capabilities including extending the keyword search to include all data types (instead of just genes); better cross-InterMine search result ordering; an offline mode with data cached in a local database for access when no internet connection is available, and a more sophisticated query construction capability for more advanced users.

## Data availability

All data underlying the results are available as part of the article and no additional source data are required.

## Software availability

**The InterMine app is available from the Google Play Store:** https://play.google.com/store/apps/details?id=org.intermine.app.

**Source code available from:** https://github.com/intermine/intermine-android .

**Archived source code at time of publication:** https://doi.org/10.5281/zenodo.1478646 ^10^.

**License:** GNU General Public License v2.
