# Supplementary material for: The InterMine Android app: Cross-organism genomic data in your pocket
Source: F1000Res. 2019 Jun 7;7:1837. Originally published 2018 Nov 22. [Version 2] doi: 10.12688/f1000research.17005.2 (PMC6572867; doi:10.12688/f1000research.17005.2)
Supplement: Peer review history

# Peer review: Reviewer response for version 2

**DOI:** 10.5256/f1000research.21313.r49658

**Published:** 2019-06-14

**Authors:** Zeeshan Ahmed

**Affiliations:** 1 University of Connecticut Health Center, Farmington, CT, USA

I have read this submission. I believe that I have an appropriate level of expertise to confirm that it is of an acceptable scientific standard.

# Peer review: Reviewer response for version 1

**DOI:** 10.5256/f1000research.18593.r43497

**Published:** 2019-02-18

**Authors:** Zeeshan Ahmed

**Affiliations:** 1 University of Connecticut Health Center, Farmington, CT, USA

Authors have produced a smart phone app, and I would like to congratulate them for good work.

 Here, I provide some comments:

 Introduction is brief; there is no background and rational provided, which can justify the need of such application. Moreover, there are no details provided of similar smart phone applications and other related platforms (e.g. web, desktop etc.). I would suggest to address these points comprehensively and add comparative analysis of their app with other related applications, including a table based on common and variable features would be helpful.

 As authors are interested in publishing their app as a scientific contribution, it’s important to have scientific justifications and discussion. At this time paper is more like a brief report.

 Methodology; why Android based smart phone app, why not iOS?

 Is data behind the app (collection of databases) freely accessible to the users, so they can download, and even verify the results and with other referenced databases? If not, then mention it in the paper, and give reasons for that. As this is an open source work, and data is collected from multiple open sources, its expected to have access to the data linked at the backend.

 Authors have mentioned list of databases, it’s important to mention licensing information of those database, to avoid any conflict of interest. Moreover, its important to clearly mention it in the conflict of interest section.

 Author’s contribution are missing, it’s also important to list those.

 I would suggest to write supplementary material and there explain the app in detail (step-by-step). Guide a new user as to how to get access to the app and how each interface can be used, and what are expected inputs/outputs. Furthermore, if word count restriction does not allow, then further extend supplementary material and provide comprehensive details of software implementation, database design and data workflow with rational for choosing those options. Make some diagrams (based on software engineering concepts) explaining design and implementation parts.

 I would suggest authors to also mention:

1. Current limitations of the app.
2. Current advantages of the app, which signifies it technically and scientifically.
3. What were the major technical, non-technical, and scientific difficulties they have faced while designing and developing this app.
4. Future recommendations, in their view and for other readers.

Regarding Figures; Figure 1 seems isolated. I would rather suggest make one good multi panel Figure, and add 1, 2, 3, 4 to that.

I have read this submission. I believe that I have an appropriate level of expertise to confirm that it is of an acceptable scientific standard, however I have significant reservations, as outlined above.

# Response

**Published:** 2019-05-22

**Authors:** Rachel Lyne

**Affiliations:** Dr, UK

**Authors have produced a smart phone app, and I would like to congratulate them for good work.**

We thank the reviewer for his detailed comments on the manuscript. We have made several changes to the manuscript and have made further comments below which we hope address the points made:

**Here, I provide some comments:**

**Introduction is brief; there is no background and rational provided, which can justify the need of such application. Moreover, there are no details provided of similar smart phone applications and other related platforms (e.g. web, desktop etc.). I would suggest to address these points comprehensively and add comparative analysis of their app with other related applications, including a table based on common and variable features would be helpful.**

**As authors are interested in publishing their app as a scientific contribution, it’s important to have scientific justifications and discussion. At this time paper is more like a brief report.**

As the development of mobile apps for biology is still in its infancy and as this provided a new and novel way of accessing the data in InterMine, it’s development was largely exploratory as we did not know at the outset how much demand for the app there would be. The very popular iOS app developed by the Saccharomyces Genome database (utilizing their yeastMine webservices) suggested that this could become a popular way in which data could be accessed (Wong et al; PMID: 23396302). Indeed, the InterMine app has already had well over one hundred downloads and we hope that with continued development this number will increase. We have added a paragraph to the introduction to cover these points.

**Methodology; why Android based smart phone app, why not iOS?**

An iOS app has been developed independently and will be published separately as the technology is quite different.

**Is data behind the app (collection of databases) freely accessible to the users, so they can download, and even verify the results and with other referenced databases? If not, then mention it in the paper, and give reasons for that. As this is an open source work, and data is collected from multiple open sources, its expected to have access to the data linked at the backend.**

Search results from within the app can be shared as described under “Search” in the Implementation section. Further export options are available if the full InterMine database is accessed via the app.

We have added a sentence to under “search” of the Implementation section to make this clearer to the reader.

**Authors have mentioned list of databases, it’s important to mention licensing information of those database, to avoid any conflict of interest. Moreover, its important to clearly mention it in the conflict of interest section.**

All the InterMine databases are open source and provide open access to the data within them.  

However there are some restrictions for commercial use of some of the data and each individual InterMine should be consulted for its policy. We have added a sentence to the methods section to make this clearer to the reader.

**Author’s contribution are missing, it’s also important to list those.**

Author contributions were provided in the original paper and are available under the author details.

**I would suggest to write supplementary material and there explain the app in detail (step-by-step). Guide a new user as to how to get access to the app and how each interface can be used, and what are expected inputs/outputs. Furthermore, if word count restriction does not allow, then further extend supplementary material and provide comprehensive details of software implementation, database design and data workflow with rational for choosing those options. Make some diagrams (based on software engineering concepts) explaining design and implementation parts.**

We feel that the three key aspects of the interface have been fully explained through the use-case and accompanying screenshots. Further details for all aspects of the InterMine interface can be found in previous publications. The InterMine database design and the webservices used to power the InterMine app have also been previously described. The following sentences have been added to the manuscript to direct readers more readily to this information:

Introduction (to use-case)

The following use case introduces each of the three key features of the InterMine app; Gene search, Templates and Lists with an example of their use.  Further details of these InterMine features have been fully described in previous publications from several InterMine databases (1,2,3,5,6,8,9).

Added as first sentence under Communication:

The InterMine database design and the webservices used to power the InterMine app have been previously described (1, 14).

# Peer review: Reviewer response for version 1

**DOI:** 10.5256/f1000research.18593.r42072

**Published:** 2019-01-10

**Authors:** Johan Nyström-Persson

**Affiliations:** 1 Lifematics Co. Ltd, Tokyo, Japan

The authors describe the InterMine Android app, which makes data available from InterMine applications on Android devices. Significantly, it is able to combine data from multiple data warehouses, and users can add their own InterMine instances to this list.

 The source code is GPL licensed and available on GitHub, and the source code as it was at the time of publication has also been archived.

 For this review, unfortunately I did not have access to an Android device, so I assume that the application functions as described.

 The app seems to contain a carefully chosen set of core features to enable quick searches, for example by gene or template, and presents combined search results in a streamlined user interface. It is also possible to access gene lists. The WebView feature to display visualisations should also be useful, although most likely the user experience here is harder to control by nature (owing to the different widgets that may be available in InterMine instances being accessed).

 I am curious as to how the app would behave if one queries different InterMine instances that have very different response times and displays the combined results. Hopefully, the app would display results incrementally as they are available, instead of waiting for the slowest instance to reply and then combining the results.

 The authors have also taken reasonable care to handle authentication details and transmitted information securely.

 In summary, the InterMine Android App should be useful for querying InterMine instances on the go from handheld devices, but also for combining data from different instances in a way that, as far as I know, has not been possible until now. I recommend acceptance of this report.

I have read this submission. I believe that I have an appropriate level of expertise to confirm that it is of an acceptable scientific standard.
